# Solving User Priority in Cloud Computing Using Enhanced Optimization Algorithm in Workflow Scheduling

**DOI:** 10.1155/2022/7855532

**Published:** 2022-08-28

**Authors:** Ambika Aggarwal, Sunil Kumar, Ashutosh Bhatt, Mohd Asif Shah

**Affiliations:** ^1^School of Computer Science, University of Petroleum and Energy Studies, Dehradun 248001, India; ^2^Computer Science & Engineering, Shivalik Group of Collegers, Dehradun 248001, India; ^3^Bakhtar University, Kabul, Afghanistan

## Abstract

Cloud computing is a procedure of stockpiling as well as retrieval of data or computer services over the Internet that allows all its users to remotely access the data centers. Cloud computing provides all required services to the users, but every platform has its share of pros and cons, and another major problem in the cloud is task scheduling or workflow scheduling. Multiple factors are becoming a challenge for scheduling in cloud computing namely the heterogeneity of resources, tasks, and user priority. User priority has been encountered as the most challenging problem during the last decade as the number of users is increasing worldwide. This issue has been resolved by an advanced encryption standard (AES) algorithm, which decreases the response time and execution delay of the user-request. There are multifarious tasks, for instance, deploying the data on the cloud, that will be executed according to first come first serve (FCFS) and not on the payment basis, which provides an ease to the users. These investigated techniques are 30.21%, 25.20%, 25.30%, 30.25%, 24.26%, and 36.9 8% improved in comparison with the traditional FFOA, DE, ABC, PSO, GA, and ETC, respectively. Moreover, during iteration number 5, this approach is 15.20%, 20.22%, 30.56%, 26.30%, and 36.23% improved than that of the traditional techniques FFOA, DE, ABC, PSO, GA, and ETC, respectively. This investigated method is more efficient and applicable in certain arenas where user priority is the primary concern and can offer all the required services to the users without any interruption.

## 1. Introduction

Cloud computing technology is a perfect recreation to build and run multifarious applications in the modern world, where these applications are executed as utilities instead of just a piece of software that runs on the desktop of a user over the Internet [1]. Cloud computing may be described as a variation of computing services executed through the Internet. The services of cloud computing often include servers, networks, databases, software, analytics, and many more computing functions, which are executed over the cloud [2].

### 1.1. Cloud Services

**Figure 1:**
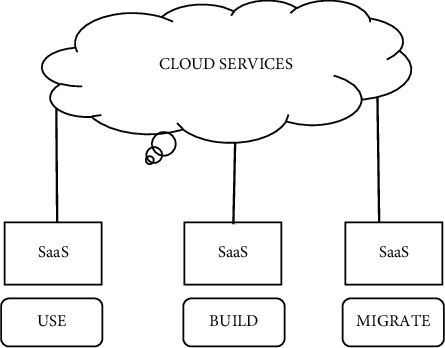
Cloud services offered by cloud computing platforms.

Cloud computing delivers numerous services and these services are fragmented into three different categories that are Software-as-a-Service (SaaS), Platform-as-a-Service (PaaS), and Infrastructure-as-a-Service (IaaS) as depicted in Figure 1 [3]. SaaS is one of the most common services that is used daily and it dispenses all functions of traditional applications to many users through World Wide Web (WWW), which is not a locally installed application. There are multifarious services, for instance, Google (Gmail and alternative applications such as docs), salesforce, and skype. PaaS sends virtualized servers on which clients may execute and deploy web applications deprived of concern about the operating system, server hardware, and balancing of the load. This renders full support to a full life cycle of applications, which assists multiple users to test, manage, update, and many more according to the user's demands. These services also include various development tools, business intelligence solutions, and middleware. There are certain well-known service providers, e.g., PaaS is being offered by azure, force.com, AWS elastic beanstalk, and Google maps. IaaS offers basic computer infrastructure services that provide data storage, servers, and hardware to users all over the cloud. It also provides business access to large platforms and applications without any huge on-site physical infrastructure. There are numerous vendors of IaaS including Amazon, EC2, and Google Compute Engine.

There are numerous attributes of cloud computing such as self-service on request, wide network accessibility, pooling of resources, quick elasticity as well as measured services [4]. The self-service on request platform user may access several computing proficiencies namely the time of server as well as network storage that is demanded spontaneously without human intervention. On the other hand, wide network access provides numerous computing services that are vacant over the network and may be reached via retrieved mechanisms such as mobile phones, laptops, and tablets. In the pooling of the resources, the provider's computing resources can be pooled to serve multifarious clients by utilizing a multitenant architecture with diverse physical as well as virtual resources that are assigned animatedly and reassigned as per the customer request. The rapid elasticity provides ease to the users because they can buy services of cloud computing according to their requirements and the measured services spontaneously control and enhance resource employment by leveraging a metering ability that is suitable to the kind of facility, for instance, storing, processing, routing, and bandwidth [5].

### 1.2. Cloud Infrastructure

The cloud infrastructure comprises of software and hardware components such as servers, virtualization, and network. The infrastructure of cloud computing is categorized into four different classes namely public cloud, private cloud, community cloud, and hybrid cloud [6].

The private cloud is solely functioning to be used by a particular organization and various services such as security and authentication, and availability of resources are provided to particular private organizations. The private cloud offers high security and privacy with the help of the firewall and the Internet host [7]. In the public cloud applications, storage and other resources can be accessible to the clients and such types of services are either free of cost or accessible according to the pay-per-use model. These public cloud applications are utilized for many purposes such as application development, e-mail, sharing of data files, and testing. There are multifarious companies that provide public cloud services namely Google, and Microsoft. Community cloud is another essential cloud infrastructure that is used for sharing the infrastructure between certain organizations from a definite community with mutual apprehensions namely the secrecy and jurisdiction, and accomplished either inside or outside or by a mediator. This cloud is advantageous for numerous applications where the infrastructure of this cloud is provisioned for exclusive use. Hybrid cloud infrastructure is very useful and has less computational complexity in comparison to others. It is a mixture of two or more clouds infrastructure namely the private community or public that remains as distinctive objects but are bounded and self-possessed. This type of cloud infrastructure has numerous advantages and applicability that resolve various existing issues in cloud computing infrastructure. These cloud infrastructures can be used by several application areas [7, 8].

### 1.3. Task Scheduling in Cloud Computing

The task scheduling in cloud computing is specifically an NP-complete issue and during this procedure, consumers acknowledge their requests to a cloud scheduler. Furthermore, schedulers inspect the accessibility of assets and their possessions in return, which assigns each request to various available resources accordingly and multiple tasks can be assigned. Cloud computing is a diverse viewpoint for a hefty scale disseminated computing as well as the parallel processing that offers computing like a utility service according to pay-per-use service. The performance, as well as the efficacy of the cloud services, permanently fall under the performance according to a client task that is given to cloud systems.

### 1.4. Task Scheduling Algorithms

Various algorithms have been explored to schedule tasks in a cloud computing environment and these approaches can be further divided into the heuristic approach, hybrid approach, and energy-efficient approach, which are explained in this section [9, 10].

#### 1.4.1. Heuristic Approach

The heuristic approach is a pragmatic way to resolve the encountered NP-complete problem and considers the knowledge base for taking all scheduling decisions [11]. These algorithms can be classified into two separate categories i.e., static and dynamic and some notable instances of these algorithms are genetic and simulated annealing algorithms. In the genetic approach, four diverse actions i.e., assessment, assortment, crossover, and mutation are performed, whereas, simulated annealing is an iterative process that may be characterized as analogous to a genetic algorithm, wherein it begins with a solitary result (mapping) nominated from random distribution method. These approaches are considered to be approximately good to some extent and not as any accurate algorithms because these approaches can find solutions among all possible domains to some extent.

#### 1.4.2. Hybrid Approach

These types of methods have been developed over some existing work by incorporating more scheduling parameters to certain current versions of algorithms and being able to enhance the performance in a more effective manner. Self-adaptive fruit fly optimization (SAFFO) is the enhanced version of an existing fruit fly algorithm used for scheduling workflows in a cloud computing environment, which is an example of a hybrid approach [12].

#### 1.4.3. Energy-Efficient Approach

Power management in cloud computing systems depends on numerous features and task scheduling is another notable factor in all of them [13]. Multiple task scheduling algorithms have been investigated to reduce the power consumption, which is used to enhance the performance parameters of the system and minimize overall cost as well. There are two techniques to minimize the consumption of energy as well as to upsurge the use of servers within various data centers. The first technique considered is to make consolidate jobs. Previously, the investigator's primary objective was to use the minimal number of servers (First-fit decreasing, FFD) that has an approximation to receive the minimum number of bins within the bin packing to attain the required power saving, which promises that the assigned workload may be completed by using the clusters.

The second approach uses a technique named dynamic voltage frequency scaling (DVFS). This investigated approach is a mixture of the dynamic voltage scaling (DVS) technique as well as dynamic frequency scaling (DFS) method. There are various methods for scheduling a task within cloud computing infrastructure and a good technique always improves CPU utilization, combined throughput, and turnaround time. Although task scheduling is not prominent enough in cloud computing and has many drawbacks. Task scheduling is facing multiple challenges due to the following features of cloud computing.

*(1) Heterogeneity of Resource*. There are many resources available in cloud computing, which are of different types such as mobiles, laptops, supercomputers, and many more and it is a huge challenge to schedule a task on these types of resources efficiently and in a very small amount of time as demanded in the modern world.

*(2) Heterogeneity of Tasks*. Different users have different requirements and they perform their tasks according to their demands and many other factors such as the available time and the urgency of any task. The tasks rendered to the cloud are miscellaneous and hence the heterogeneity of tasks depends on the user requirements.

*(3) User Priority*. Cloud computing offers better and more prominent services to users who pay more. Privileges are given on a payment basis and high priority is given to the user who pays more. This investigated approach resolves the issues of user priority by introducing an encryption method before the task scheduling algorithm. The user requests will be fetched to the cloud server in encrypted form and the cloud server will further generate a token that gives the information when the request will be scheduled. The problem of user priority has been resolved by the cloud server by using encryption requests and the user information will not be encountered by the cloud that offers secrecy and is demanded in the modern world as security of data is a huge challenge in cloud computing. Some researchers have found the solution to existing issues to some extent to determine how a task or workflow is scheduled in cloud computing and what are the impacts of scheduling on cloud platforms.

The paper organization is as follows, section 1 discusses the introduction of cloud computing, cloud services, cloud infrastructure, and various task scheduling approaches.  Section 2 represents a detailed explanation of literature reviews about task scheduling algorithms.  Section 3 shows the design for the proposed work.  Section 4 highlighted the results and discussion of the proposed work followed by the conclusion and future directions of the proposed study.

## 2. Literature Review

Bui et al. [14] investigated the workflow scheduling model that efficiently schedules tasks such that all the assigned tasks will be performed within minimal time to preserve the quality of service (QoS) and satisfy customer's demands. Workflow scheduling has been found as a major issue in cloud computing algorithms and these approaches need to be developed to improve the QoS in the modern world. The cloud computing sector is growing dramatically, and therefore, the workload has increased in multiple cloud services to handle clients' requests for a particular service. In this research paper, the authors investigated a method to resolve the existing issues of taking more time for a user request to some extent and to offer more fast services to the clients.

Singh and Petriya [15] discussed their research about how workflow scheduling, as well as resource allocation, is to be accomplished using the heuristic approach. In this paper, the authors investigated a heuristic method that combines the enhanced analytic hierarchy process (MAHP), bandwidth-aware divisible scheduling (BADS) as well as BAR optimization, longest expected processing lime preemption (LEPT), and segregate and conquer techniques to accomplish job scheduling and allocation of the resources. In this method, every assigned job is administered before its real distribution to a specified cloud resource by applying the MAHP procedure. In this research article, the authors proposed a method that is better than the conventional heuristic approach that increases the efficiency and turnaround time of a system but there is a scope for developing more efficient scheduling algorithms that enhance the performance of a system.

Patel and Bhoi [16] reviewed several priority-based task scheduling algorithms and performed their comparison so that one can conclude which algorithm is more beneficial and can be approached for further research in the sector of cloud computing to resolve the existing issues which is a huge challenge to offer the best services to the user within less time. In this literature review article, the authors have analyzed various conventional scheduling algorithms in cloud computing and found that the PA-LBIMM algorithm is the best among all priority-based algorithms. Various other researches have been conducted where optimization of many algorithms such as genetic and fruit fly has been done to get improvised results.

Patel and Bhoi [17] reviewed another technique for workflow scheduling and examined the aim of workflow scheduling and how it has become a protuberant matter in cloud computing infrastructure. One of the primary threats, that has been talked about in various workflow scheduling approaches is the idea of protected scheduling. Numerous state-of-the-art workflow scheduling methods have been investigated in this open literature, and furthermore, this research article offers a comprehensive overview of the existing issues and the proposed methods in the sector of cloud computing. There may be found certain systems that can be proposed with encryption to offer security to workflow scheduling, which considers the ever-increasing cyber-attacks.

Masderi et al. [18] provided an inclusive theoretic investigation on how the selected duplicating task precursor is supportive to prevent both data transmission time as well as the encryption time from the begining time of a postponed task. Anyone may describe the workflow scheduling latest completion time as well as demonstrate that selected tasks may be done before tasks latest finish timing by applying the inexpensive resources to minimize the overall cost deprived of any delay in tasks successor beginning time as well as the workflow scheduling makespan. In this article, the authors have devised an innovative scheduling tactic by using a discerning task duplication and encryption that triumphs over orthodox procedures in terms of makespan, monetary costs, and resource efficiency. These encryption techniques can also be used to solve challenges in workflow scheduling such as the heterogeneity of tasks, heterogeneity of resources, and user priority.

Chen et al. [19] investigated another method for the task arrangement in order to poise the overall burden in the cloud computing arena. The cloud computing infrastructure has been developed as a novel architecture of comprehensive distributed computing. Certain issues have been encountered related to load balancing and the user priority in cloud computing that demand more attention. The proposed algorithm offers the task scheduling approach that utilizes minimal makespan, but this technique is not much appropriate from the resource utilization perspective and has many issues with present systems. This proposed algorithm has been utilized widespread due to many factors such as very less computational complexity and many more. In this paper, the authors found that the proposed algorithm is very much efficient and reliable because it decreases the makespan as well as improves the utilization of selected resources in comparison to existing algorithms.

In this research paper, the AES encryption technique is used to solve the issue of user priority [20]. All the above researchers have discussed how a workflow or task scheduling executes in cloud computing. Some researchers have reviewed the various techniques for scheduling a job in cloud computing and what are the challenges faced during scheduling. Some authors have devised new algorithms to improve response time, execution delay, and maintain QoS. Few authors have included the encryption method to provide security against various cyber-attacks. In this paper, the major and huge challenge of workflow scheduling i.e., user priority has been resolved by applying the AES encryption method [21]. The encryption algorithm which is incorporated in this approach hides the identity of a user so that cloud service providers can schedule requests on a first come first serve rule and not on a priority basis. Cloud is a platform that offers services on a payment basis and the more a person pays, the faster and more efficient services will be offered. To solve this challenge this method is incorporated with a conventional workflow scheduling algorithm and offers prominent services to the clients.

## 3. Proposed Method

### 3.1. Design

This experiment is conducted in such a way that accurate and reliable results are recorded and validated with the help of the MATLAB simulator. In this paper, an encryption method is utilized before the task scheduling algorithm which is a novel approach and thus solves issues related to user priority in cloud computing. Multifarious algorithms have been investigated such as advanced encryption standard (AES), Rivest-Shamir-Adleina (RSA), data encryption standard (DES), triple data encryption standard (Triple DES), and many more. In this research paper, a conventional, reliable, and more secure AES algorithm is used for encrypting the user's requests. The AES algorithm is famous and utilized globally for the block cipher symmetric processes for encryption and it is applied over both data at rest and data in transit.

**Figure 2:**
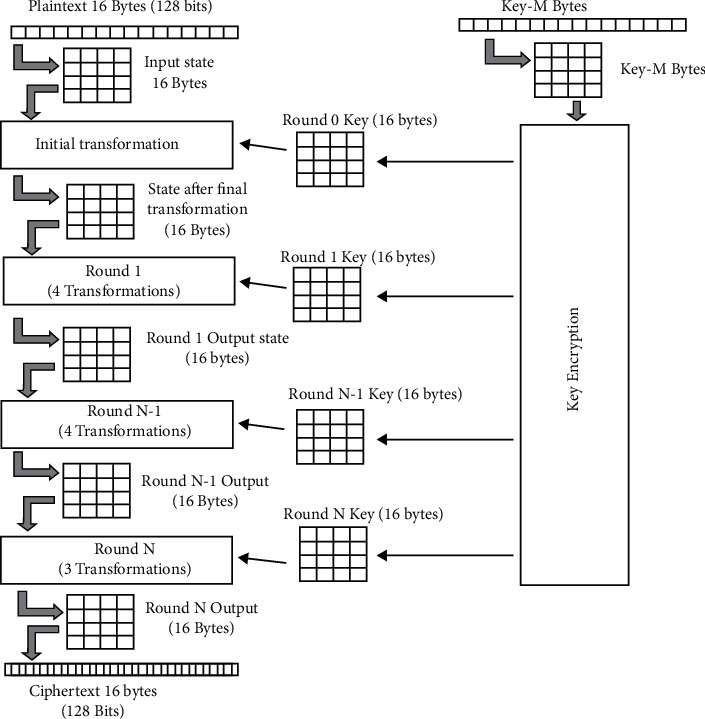
Process of advanced encryption standard (AES) and conversion of plaintext 16 bytes into various ciphertext 16 bytes using various round keys and key encryption methods.

Moreover, this AES algorithm can deal properly with diverse key extents namely AES 125, 192 as well as 256 bit, and every cipher has a block size of 128 bit. The encryption process is a method to protect the privacy of user data and for this AES algorithm uses the technique of replacement and transformation network that carries a lot of scientific operations that are accepted within block ciphers. Referring to Figure 2 the steps for the AES algorithm are given below.

  Step 1 : to develop a set of round keys from the selected cipher key
  Step 2 : to initialize the state array with the block data (plain text)
  Step 3 : to compute the primary round key to the initial state array
  Step 4 : to execute 9 rounds of state manipulation
  Step 5 : to execute the tenth and the last round of state manipulation
  Step 6 : to copy the last state array out like encrypted information (cipher)

The test was executed on the MATLAB software and the test results are observed and verified properly with high precision [22]. The MATLAB simulator offers multifarious features for the testing of multiple methods or processes and provides great flexibility in numerous applications globally. MATLAB offers an online platform as well as one can utilize MATLAB in any web browser directly without software installation or configuration, which provides huge flexibility in the modern era and provides numerous applicability for multiple testing. The simulation tool of MATLAB has multiple advantages and is applicable in numerous testing environments. Simulink online is a pragmatic platform for users because it offers access to Simulink with the help of any browser, wherein, users can sign up and can start to access Simulink or can access an open Simulink model [23].

There are numerous tools in MATLAB software that are being utilized globally for various testing purposes such as statistics and machine learning, control systems, signal processing, financial toolbox, data feed toolbox, and several other important ones for testing purposes. In this experiment, MATLAB R20 17b software package was used with a system configuration of 64-bit operating system in Windows 10 with 8 GB RAM. The test was conducted in many phases to acquire the optimum results to validate the proposed algorithm with high precision. The testing algorithm was verified and validated in a well-structured manner to reduce any redundancy and to get better results, which are demanded in the modern world. The secrecy of the data was maintained during testing and after that results were observed.

### 3.2. Data Collection

For the validation of the proposed AES algorithm, it is assumed that five different tasks have been submitted by various customers for scheduling on the availability of two diverse resources.  Table 1 shows the overall processing speed (mb/sec) for each selected resource for scheduling diverse tasks in cloud computing. The users are independent to take a variety of services according to their requirements either VIP or ordinary. This experiment was conducted in three different segments for the optimal validation of the proposed AES algorithm and results were compared after verification of the proposed AES algorithm with the load balance improved min-min scheduling algorithm (LBIMN) [24]. The LBIMN algorithm has been discussed in open literature with existing issues related to task scheduling and user priority in cloud computing.

Table 2 presents the different task sizes in MB for a variety of tasks in pursuance of the user's requirements. This table is utilized for the computation of predictable completion time and completing time of selected tasks for every selected resource. The proposed AES algorithm was tested and validated by dividing the whole test into three different segments in order to achieve minimal task completion time, enhanced load balancing as well as customer satisfaction according to their demands.

Multifarious algorithms have been presented for scheduling the tasks on the cloud but in this experiment, a self-adaptive fruit fly optimization algorithm (SAFFOA) has been used. For numerous workflow, scheduling is utilized. SAFFOA is designed by using the combination of both genetic as well as fruit fly optimization algorithms [12]. The recommended procedure is pragmatic concerning to flow interval with the smallest rate, and this proposed model is improved over the fruit fly optimization algorithm (FFOA) by 0.23%, differential evolution by 2.48%, artificial bee colony (ABC) by 2.85%, particle swarm optimization (PSO) by 2.46%, genetic algorithm (GA) by 2.33%, and expected time to compute (ETC) by 2.56%. In case of makespan analysis, the proposed method is optimal in comparison to conventional approaches namely FFOA, DE, ABC, PSO, GA, and ETC by 0.28%, 0.15%, 0.38%, 0.20%, 0.21%, and 0.29%, respectively. Moreover, the cost of this investigated model is quite low in comparison with other existing models and is enhanced by 2.14% than FFOA, 2.32% than DE, 3.53% than ABC, 2.43% than PSO, 2.07% than GA, and 2.90% than ETC.

(1) $$\begin{array}{c} O=\hat{a}*\text{Makespan}+\hat{b}*\text{flowtime}+\hat{c}*\frac{x}{r}, \end{array}$$

(2) $$\begin{array}{c} X_{i}=X_{i}+X_{i}*RV\left(0,1\right)*\left(X_{i}\right)+\bar{F}. \end{array}$$

Equation (1) represents the objective function with makespan and flowtime with arbitrary constant $\hat{a},\;\hat{b},\text{ and }\hat{c}$. The term *x*/*r* represents the random values. In equation (2), *X*_*i*_ represents the coordinate position of the fly with a mean value *F*.

Self-Adaptive Fruit Fly Optimization Algorithm.

**Step 1 .**

Initialize parameters maximum population, and initial locations.

**Step 2 .**

Evaluate the fitness by applying (1)

**Step 3 .**

Till the stopping criteria are met

Randomly select location through distance and smell concentration judgment value.

Update every fruit fly by applying (2).

Evaluate fruit fly with maximal small concentration.

Rank the solutions and provide the best solution.

**Step 4 .**

Postprocess and observe the result.

**Figure 3:**
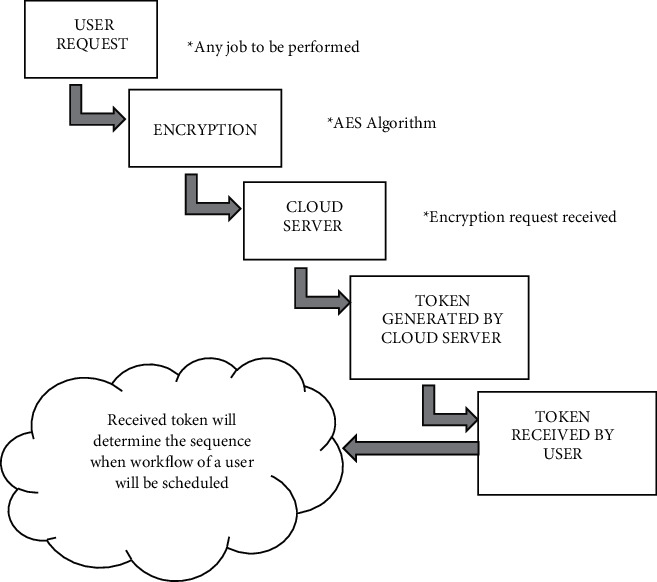
Workflow scheduling in cloud computing incorporated with advanced standard encryption (AES).

The above algorithm makes the workflow scheduling process more efficient and reliable but the problem of user priority is still prominent [12]. To resolve this existing problem an encryption model is applied before the process of workflow scheduling. As cloud computing is based on payment methods, the user's first demand is to pay his share (money) for all the services the user wants to make use of, and after the successful completion of payment, the user can uninterruptedly enjoy the services on the cloud platform. Referring to Figure 3 the existing issues can be resolved by applying the below approach.

  Step 1 user payment for accessing services on the cloud platform
  Step 2 after successful payment, the user requests his workflow schedule
  Step 3 workflow schedule request is encrypted using AES encryption algorithm
  Step 4 the encrypted request is fetched to the cloud server
  Step 5the cloud server on receiving encoded requests generates tokens
  Step 6 tokens are further rendered to the user
  Step 7 the rendered token will generate information to the user when his request will be scheduled without any partiality based on money payments as the cloud server will receive encrypted requests that will not disclose the identity of a particular user and regardless of even less payment his requests can be scheduled on time.

Figure 3 depicts the workflow scheduling in the cloud computing environment with AES technique to resolve the major challenge of workflow scheduling i.e., user priority. The AES algorithm is the most popular and adopted algorithm and a pragmatic solution to the issues related to workflow scheduling in the modern era.

## 4. Results and Discussion

The investigational testing of the proposed AES algorithm has been executed in three different segments. The proposed AES algorithm is a popular and suitable algorithm that provides a pragmatic solution to the threats that are associated with workflow scheduling in the modern era globally.

- Segment A: small proportion of VIP tasks
- Segment B: large proportion of VIP tasks
- Segment C: diverse numbers of unplanned tasks

For both segment A as well as B the number of selected resources and tasks were 5 and 10, respectively, for the diverse proportion of the VIP jobs. For segment C, the number of unplanned resources was selected as 50. For the optimal validation and evaluation of the test algorithm, six different numbers of unplanned tasks were selected: 100, 200, 300, 400, 500, and 600.

### 4.1. Segment A

For segment A, the total number of resources and tasks were selected as 5 and 10, respectively, in order to validate the test results. The whole test was conducted according to the two different services VIP or ordinary. This proposed AES algorithm utilizes different execution speeds according to the selected resources as shown in Table 3. Segment A has a small proportion of VIP tasks for the test verification. The users can take a variety of services as VIP or ordinary according to the requirements. For the validation of the proposed AES algorithm five resources were selected for the results verification.  Table 4 shows the task specifications for the validation of the proposed AES algorithm in Segment A. For the verification of the AES algorithm, ten different tasks were assigned by a variety of users according to their requirements.

### 4.2. Segment B

For segment B, the total number of resources and tasks were selected as 5 and 10, respectively, for the verification of the proposed AES algorithm. The whole test was conducted according to the two different services as VIP or ordinary for Segment B. This proposed AES algorithm utilizes different execution speed according to the selected resources as shown in Table 5. Segment B has a large proportion of VIP tasks for the test verification. The users can take a variety of services as VIP or ordinary according to the requirements. For the validation of the proposed AES algorithm five resources were selected for the results verification.  Table 6 depicts the task specifications for the validation of the proposed AES algorithm in Segment B. For the verification of the AES algorithm, ten different tasks were assigned by a variety of users according to their requirements.  Table 7 illustrates the performance results of segment A for the proposed AES algorithm as well as the LBIMN algorithm for the optimal calculation and verification of different performance parameters. The observed makespan, average VIP job accomplishment time (sec), and the average conventional task completion time (sec) were measured optimal by using the proposed AES algorithm.  Table 8 illustrates the performance results of segment B for the proposed AES algorithm as well as the LBIMN algorithm for the optimal calculation and verification of different performance parameters. The observed makespan, average VIP task completion time (sec), and the average ordinary task completion time (sec) were measured optimal by using the proposed AES algorithm.

### 4.3. Segment C

The diverse numbers of unplanned tasks were selected for the verification of the proposed AES algorithm. For the optimal validation and evaluation of the test algorithm, six different unplanned tasks were selected: 100, 200, 300, 400, 500, and 600.  Table 9 illustrates the random task and resource specification for the selected tasks for segment C. For the optimal validation and evaluation of the proposed test algorithm, six different unplanned tasks were selected as shown below.

**Figure 4:**
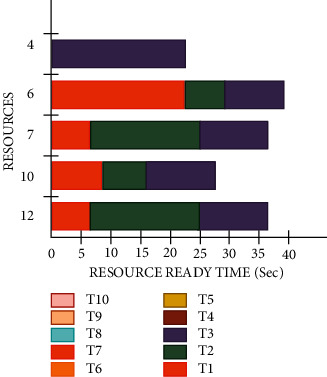
Gantt chart for the proposed AES algorithm for selected tasks for segment A.

Figure 4 illustrates the Gantt chart for the proposed AES algorithm for selected tasks for segment (A) The proposed AES algorithm provides the minimal average of the VIP tasks completion time (seconds), makespan, and average conventional task end time (seconds).

**Figure 5:**
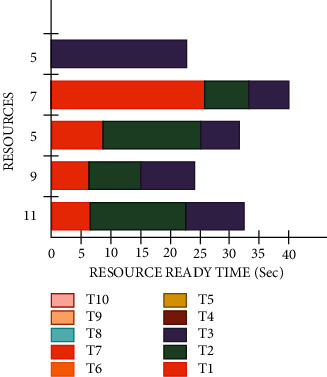
Gantt chart for the proposed AES algorithm for selected tasks for segment B.

Figure 5 illustrates the Gantt chart for the proposed AES algorithm for selected tasks for segment (B) The proposed AES algorithm provides the minimal average VIP task ending time (seconds), makespan, and average conventional task ending time (seconds).

The user's priority is one of the most challenging issues in workflow scheduling in the cloud computing sector and numerous research has been conducted to resolve this issue. In this research paper, a novel approach has been investigated for a pragmatic and optimal solution, wherein an encryption method is applied before the workflow scheduling in cloud computing. This encryption process was done with the help of the AES algorithm because this encryption method provides security on the user's request for performing a job on the cloud and hides the identity of a user. This encrypted request is scheduled without any partiality and decreases the response time for execution of a job to provide a smooth working to the user without any delay and follow the rule of OCMS. This investigated approach is more suitable, efficient, and reliable and takes less time in comparison to existing methods to resolve the issue of the user priority. During the test, certain parameters were measured such as the request time for each user was 0.5 seconds, for both internal as well as external users, the computational time was reduced by 20% for the present system model. This investigated approach is 30.21%, 25.20%, 25.30%, 30.25%, 24.26%, and 36.98% pragmatic to the traditional FFOA, DE, ABC, PSO, GA, and ETC, respectively. Furthermore, for iteration number 5, this investigated approach is 15.20%, 20.22%, 30.56%, 26.30%, 22.32%, and 36.23% pragmatic than that of the traditional methods FFOA, DE, ABC, PSO, GA, and ETC, respectively. Figure 4 shows the Gantt chart for the proposed AES algorithm for selected tasks for segment A. The proposed AES algorithm provides the minimal average VIP tasks accomplishment time (seconds), makespan, and average ordinary task completion time (seconds) of 6.15, 15.7, and 8.12 seconds, respectively, in comparison to the LBINM algorithm as mentioned in Table 7. Figure 5 shows the Gantt chart for the proposed AES algorithm for selected tasks for segment B. The proposed AES algorithm provides the minimal average VIP tasks completion time (Sec), makespan, and average ordinary task completion time (Sec) of 5.23 sec, 10.7 sec, and 5.48 sec, respectively, in comparison to the LBINM algorithm as mentioned in Table 8. The unsystematic jobs and the specification of resources for the selected tasks for segment C are shown in Table 9. For the optimal validation and evaluation of the proposed test algorithm, six different numbers of unplanned tasks were selected and test results were found pragmatic for the proposed AES algorithm.

## 5. Conclusion and Future Directions

The main motive of this research was to overcome the challenges of inequity offered by cloud computing to minimize the response time of tasks assigned by each user in cloud infrastructure. Various methods have been investigated to resolve this issue in previous years but in this experiment, the SAFFOA algorithm is utilized for diverse workflow scheduling within the cloud computing infrastructure. In this research work, an encryption technique is used before the selected task scheduling algorithm, which is a new method and efficiently resolves the issues related to user priority in cloud computing in comparison to conventional approaches. The users often need to pay money to render services from a particular cloud platform and these services can be either storing, deploying, developing websites, etc., and after the completion of the payment, the user can enjoy his share of services. Cloud computing is based on the user's priority, for instance, who pays more for the services, that user's job will be scheduled first, and due to this users face the problem of execution delay.

The execution delay is a prominent problem in both operating systems as well as cloud computing platforms and to overcome this issue an encryption system has been invented, which was applied before the workflow scheduling algorithm. Once the user requests for services, this request will be fetched into an encryption system and encryption will take place with the AES algorithm. This encrypted request is further transferred to the cloud server, which will generate a token describing the sequence number when the request will be scheduled. This investigated approach is more suitable and efficient to resolve the issues of user's priority and follows the rules of OCMS, which is the user's top priority. This investigated approach is 30.21%, 25.20%, 25.30%, 30.25%, 24.26%, and 36.98% better than that of the ordinary FFOA, DE, ABC, PSO, GA, and ETC, respectively. Furthermore, for iteration number 5, this investigated method is 15.20%, 20.22%, 30.56%, 26.30%, 22.32%, and 36.23% better than that of the traditional techniques FFOA, DE, ABC, PSO, GA, and ETC, respectively. The proposed AES algorithm offers the minimal average VIP tasks completion time (sec), makespan, and average ordinary task completion time (sec) of 6.15 sec, I 5.7 sec, and 8.1 2 sec, respectively, in comparison to the LBINM algorithm for segment A. Moreover, the proposed AES algorithm delivers the minimum average VIP task completion time (sec), makespan, and average ordinary task completion time (sec) of 5.23 sec, 10.7 sec, and 5.48 sec, respectively, for segment B. For the optimal validation and evaluation of the proposed test algorithm, six different unplanned tasks were selected and test results were found pragmatic for the proposed AES algorithm for segment C.

There is a huge scope in the sector of cloud computing to find out the best solutions to the existing issues by doing more experiments. The cloud [25] computing sector is facing multiple issues during the last decade as the number of users is increasing dramatically worldwide, which takes more time to offer optimal services within less time which is the top priority of the users. it has been observed from the conducted experiment that this scheme is more suitable, reliable, and fast in comparison to the existing approaches and offers pragmatic services to the users according to their demand, however, more research is demanded to explore more possibilities to resolve existing issues.

## Figures and Tables

**Table 1:** Processing speed for each selected resource for scheduling the tasks on cloud.

S. No.	Selected resources	Speed of processing (mb/second)	Service level
1	R1	15	VIP
2	R2	12	VIP
3	R3	9	Ordinary

**Table 2:** Task sizes in MB according to the user's requirements.

S. No.	Selected tasks	Size of the tasks (MB)	Group of users
1	T1	16	Ordinary
2	T2	22	Ordinary
3	T3	27	VIP
4	T4	32	Ordinary
*5*	T5	52	VIP

**Table 3:** Selected resources corresponding to the processing speed in mb/sec for segment A.

S. No	Sheeted resources	Speed of processing (mb/second)	Service level
1	R1	12	VIP
2	R2	10	Ordinary
3	R3	7	Ordinary
4	R4	6	Ordinary
5	R5	4	Ordinary

**Table 4:** Task specifications for the validation of the proposed AES algorithm in Segment A.

S. No.	Selected tasks	Size of the tasks (MB)	Group of users
1	T1	93	VIP
2	T2	23	VIP
3	T3	60	VIP
4	T4	48	Ordinary
5	T5	88	Ordinary
6	T6	75	Ordinary
7	T7	45	Ordinary
8	T8	2	Ordinary
9	T9	81	Ordinary
10	T10	44	Ordinary

**Table 5:** Selected resources corresponding to the processing speed in mb/sec for segment B.

S. No	Selected resources	Speed of processing (MB/Second)	Service level
1	R1	11	VIP
2	R2	9	VIP
3	R3	5	Ordinary
4	R4	7	Ordinary
5	R3	5	Ordinary

**Table 6:** Task specifications for the validation of the proposed AES algorithm in Segment B.

S. No	Selected resources	Speed of processing (mb/second)	Service level
1	T1	15	VIP
2	T2	16	VIP
3	T3	31	VIP
4	T4	3	VIP
5	T5	35	VIP
6	T6	3	VIP
7	T7	75	VIP
8	T8	98	Ordinary
9	T9	I0	Ordinary
10	Tl0	60	Ordinary

**Table 7:** Performance results of segment A for the proposed AES algorithm as well as the LBIMN algorithm for the optimal calculation and verification of different performance parameters.

S. No	Selected resources	Speed of processing (mb/second)	Service level
1	Selected tasks	10	10
2	Proportion of the VIP tasks	26%	26%
3	Selected resources	5	5
4	Proportion of the VIP resources	26%	26%
5	Makespan (second)	18	15.7
6	Average resource utilization ratio (second)	89.48%	91.74%
7	Average VIP tasks completion time	10.2	6.15
8	Average ordinary task completion time (second)	10.4	5.12

**Table 8:** Performance results of segment B for the proposed AES algorithm as well as the LBIMN algorithm for the optimal calculation and verification of different performance parameters.

S. No.	Task scheduling algorithm	LBIMN	Proposed AES
1	Selected tasks	10	10
2	Proportion of the VIP tasks	85%	85%
3	Selected resources	5	5
4	Proportion of the VIP resources	26%	26%
5	Makespan (second)	12.5	10.7
6	Average resource utilization ratio (second)	82.29%	95.74%
7	Average VIP tasks completion time	6.29	5.23
8	Average ordinary task completion time (second)	6.35	5.48

**Table 9:** Random task and resource specification for the selected tasks for segment C.

S. No	Number of tasks	100	200	300	400	500
1	Proportion of VIP tasks	45	55	45	40	20
2	No. of resources	55	55	55	55	55
3	Proportion of VIP resources	50	20	40	45	35

## Data Availability

The data used to support the findings of this study are available from the corresponding author upon request.
